# DeepG4: A deep learning approach to predict cell-type specific active G-quadruplex regions

**DOI:** 10.1371/journal.pcbi.1009308

**Published:** 2021-08-12

**Authors:** Vincent Rocher, Matthieu Genais, Elissar Nassereddine, Raphael Mourad

**Affiliations:** 1 Molecular, Cellular and Developmental biology department (MCD), Centre de Biologie Intégrative (CBI), University of Toulouse, CNRS, UPS, Toulouse, France; 2 Centre de Recherches en Cancérologie de Toulouse (CRCT), INSERM U1037, Toulouse, France; New York University, UNITED STATES

## Abstract

DNA is a complex molecule carrying the instructions an organism needs to develop, live and reproduce. In 1953, Watson and Crick discovered that DNA is composed of two chains forming a double-helix. Later on, other structures of DNA were discovered and shown to play important roles in the cell, in particular G-quadruplex (G4). Following genome sequencing, several bioinformatic algorithms were developed to map G4s in vitro based on a canonical sequence motif, G-richness and G-skewness or alternatively sequence features including k-mers, and more recently machine/deep learning. Recently, new sequencing techniques were developed to map G4s in vitro (G4-seq) and G4s in vivo (G4 ChIP-seq) at few hundred base resolution. Here, we propose a novel convolutional neural network (DeepG4) to map cell-type specific active G4 regions (*e.g*. regions within which G4s form both in vitro and in vivo). DeepG4 is very accurate to predict active G4 regions in different cell types. Moreover, DeepG4 identifies key DNA motifs that are predictive of G4 region activity. We found that such motifs do not follow a very flexible sequence pattern as current algorithms seek for. Instead, active G4 regions are determined by numerous specific motifs. Moreover, among those motifs, we identified known transcription factors (TFs) which could play important roles in G4 activity by contributing either directly to G4 structures themselves or indirectly by participating in G4 formation in the vicinity. In addition, we used DeepG4 to predict active G4 regions in a large number of tissues and cancers, thereby providing a comprehensive resource for researchers.

Availability: https://github.com/morphos30/DeepG4.

## Author summary

DNA is a molecule carrying genetic information and found in all living cells. In 1953, Watson and Crick found that DNA has a double helix structure. However, other DNA structures were later identified, and most notably, G-quadruplex (G4). In 2000, the Human Genome Project revealed the widespread presence of G4s in the genome using algorithms. To date, all G4 mapping algorithms were developed to map G4s on naked DNA, without knowing if they could be formed in a given cell type. Here, we designed a novel artificial intelligence algorithm that could map G4 regions active in the cell from the DNA sequence and chromatin accessibility. Moreover, we identified key transcriptional factor motifs that could explain G4 activity depending on cell type. Lastly, we used our new algorithm to map active G4 regions in multiple tissues and cancers as a comprehensive resource for the G4 community.

## Introduction

Deoxyribonucleic acid (DNA) is a complex molecule carrying genetic instructions for the development, functioning, growth and reproduction of all known living beings and numerous viruses. In 1953, Watson and Crick discovered that DNA is composed of two chains forming a double-helix [1]. However, other structures of DNA were discovered later and shown to play important roles in the cell. Among those structures, G-quadruplex (G4) was discovered in the late 80’s [2]. G4 sequence contains four continuous stretches of guanines [3]. Four guanines can be held together by Hoogsteen hydrogen bonding to form a square planar structure called a guanine tetrad (G-quartets). Two or more G-quartets can stack to form a G4 [3]. The quadruplex structure is further stabilized by the presence of a cation, especially potassium, which sits in a central channel between each pair of tetrads [4]. G4 can be formed of DNA [5] or RNA [6].

G4s were found enriched in gene promoters, DNA replication origins and telomeric sequences [5, 7]. Accordingly, numerous works suggest that G4 structures can regulate several essential processes in the cell, such as gene transcription, DNA replication, DNA repair, telomere stability and V(D)J recombination [5]. For instance, in mammals, telomeric DNA consists of TTAGGG repeats [8]. They can form G4 structures that inhibit telomerase activity responsible for maintaining length of telomeres and are associated with most cancers [9, 10]. G4s can also regulate gene expression such as for MYC oncogene where inhibition of the activity of NM23-H2 molecules, that bind to the G4, silences gene expression [11]. Moreover, G4s are also fragile sites and prone to DNA double-strand breaks [12]. Accordingly, G4s are highly suspected to be implicated in human diseases such as cancer or neurological/psychiatric disorders [13–15].

Following the Human Genome project [16], computational algorithms were developed to predict the location of G4 sequence motifs in the human genome [17, 18]. First algorithms consisted in finding all occurrences of the canonical motif *G*_3+_ *N*_1−7_ *G*_3+_ *N*_1−7_ *G*_3+_ *N*_1−7_ *G*_3+_, or the corresponding C-rich motif (quadparser algorithm) [19, 20]. Using this canonical motif, over 370 thousand G4s were found in the human genome. Nonetheless, such pattern matching algorithms lacked flexibility to accomodate for possible divergences from the canonical pattern. To tackle this issue, novel score-based approaches were developed to compute G4 propensity score by quantifying G-richness and G-skewness (G4Hunter algorithm) [21], or by summing the binding affinities of smaller regions within the G4 and penalizing with the destabilizing effect of loops (pqsfinder algorithm) [22]. Recently, new sequencing techniques were developed to map G4s in vitro (G4-seq) [23], and G4s in vivo (G4 ChIP-seq) [24] as regions of few hundred bases. Machine and deep learning methods were proposed to predict such G4 regions, *i.e*. regions comprising the G4(s) along with flanking sequences. For instance, Quadron—a machine learning approach—was proposed to predict G4s based on sequence features (such as k-mer occurrences) from a region of more than 100 bases, and trained using in vitro G4 regions with G4-seq [25]. By combining with regular expressions, Quadron could predict if a region was found in vitro, but also the exact location and stability value of G4(s) within the region. Other deep learning approaches had lower resolution for mapping G4s (around 200 bases), but they showed higher prediction performance. PENGUINN, a deep convolutional neural network (CNN), was trained to predict G4 regions in vitro [26]. Another CNN, G4detector, was also designed to predict G4 regions forming in vitro [27]. Thus, all current approaches aimed to predict G4 regions forming in vitro, but were not designed to assess the ability of G4 sequences to form in vivo (*e.g*. G4 activity).

Here, we propose a novel method, named DeepG4, aimed to predict cell-type specific active G4 regions (regions that were mapped both in vitro and in vivo in a given cell type) from DNA sequence and chromatin accessibility. DeepG4 implements a CNN which is trained using a combination of genome-wide in vitro (G4-seq) and in vivo (G4 ChIP-seq) peak DNA sequences, together with chromatin accessibility measures (*e.g*. ATAC-seq). For this purpose, DeepG4 exploits the genomic context (a 201-base region) of a G4, which comprises the potential G4 forming sequence, but also other DNA motifs that may play a role in G4 activity. Moreover, adding chromatin accessibility, which is publicly available for most cell lines, tissues and cancers, into the model allows to predict G4 regions that are active depending on the cell-type, since it was previously shown that in vivo G4 peaks strongly colocalize (98%) with regions identified by either FAIRE-seq or ATAC-seq, or both [28]. DeepG4 achieves excellent accuracy at predicting cell-type specific active G4 regions (area under the receiver operating characteristic curve or AUROC > 0.98). Moreover, DeepG4 identifies key DNA motifs that are predictive of active G4 regions. Among those motifs, we found specific motifs resembling the G4 canonical motif (or parts of G4 canonical motif), but also numerous known transcription factors which could play important roles in enhancing or inhibiting G4 activity directly or indirectly. By mapping active G4 regions that encapsulate one or more potential G4s, DeepG4 represents a complementary approach to existing algorithms based on regular expressions or propensity scores, which can be further used to precisely localize the G4s within the active G4 regions.

## Materials and methods

### G4 data

We downloaded G4 ChIP-seq data for HaCaT, K562 and HEKnp cell lines from Gene Expression Omnibus (GEO) accession numbers GSE76688, GSE99205 and GSE107690 [24, 28, 29]. For every cell line, replicates were mapped to hg19 and merged for peak calling using macs2 with default parameters (https://pypi.org/project/MACS2/). We downloaded G4P ChIP-seq (similar to G4 ChIP-seq) peaks already mapped to hg19 for A549, H1975, 293T and HeLa-S3 cell lines from GEO accession number GSE133379 [30]. We used peaks from both replicates (when there were two available replicates). We downloaded processed G4-seq peaks mapped to hg19 from GEO accession number GSE63874 [23]. We used G4-seq from the sodium (Na) and potassium (K) conditions. No filtering step was performed on peak selection.

### Active G4 sequences

We defined positive DNA sequences (active G4 region sequences) as forming both in vitro and in vivo G4s as follows. We only kept G4 ChIP-seq peaks overlapping with G4-seq peaks. We then used the 201-bp DNA sequences centered on the G4 ChIP-seq peak summits.

As negative (control) sequences, we used sequences randomly drawn from the human genome with sizes, GC content (% GC), and repeat content (tandem repeat number from Tandem Repeat Finder mask from hg19 genome) similar to those of positive DNA sequences using genNullSeqs function from gkmSVM R package (https://cran.r-project.org/web/packages/gkmSVM).

### Chromatin accessibility

We downloaded processed DNase-seq bigwig files for different cell lines from ENCODE [31], and processed ATAC-seq bigwig files for HaCaT cell line from GSE7668. We downloaded processed ATAC-seq bigwig files from ICGC cancer cohorts from https://gdc.cancer.gov/about-data/publications/ATACseq-AWG [32].

### ChromHMM annotations

We downloaded ChromHMM annotations for ENCODE cell lines from http://hgdownload.cse.ucsc.edu/goldenpath-hg19/encodeDCC/wgEncodeBroadHmm/ [33].

### BRCA cancer mutations

We downloaded breast cancer processed mutation data from ICGC BRCA-US cohort from the portal https://dcc.icgc.org.

### JASPAR DNA motifs

We used position weight matrices (PWMs) for transcription factor binding sites from the JASPAR 2018 database (http://jaspar.genereg.net).

### DeepG4 model

**Fig 1:**
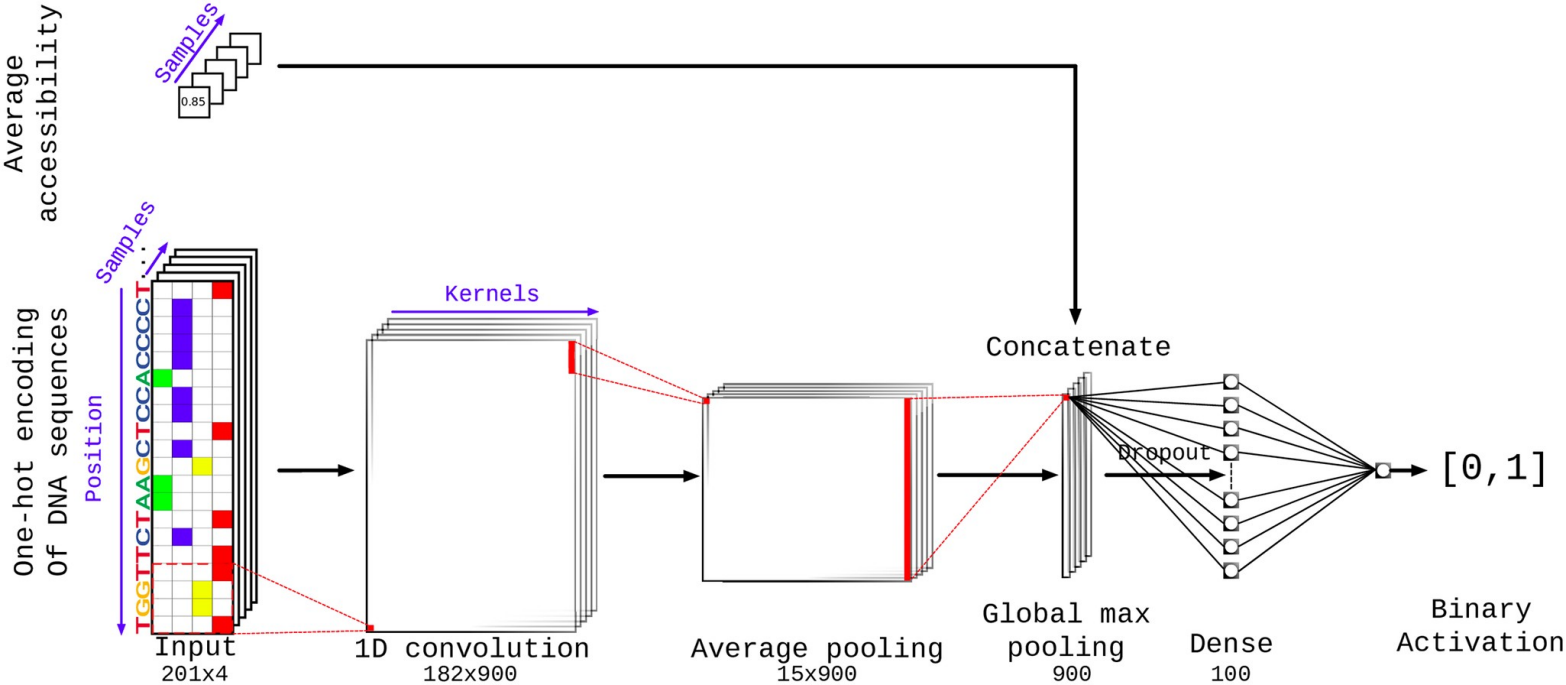
DeepG4 model architecture. Here, one-hot encoding is a numerical encoding of a 201-bp DNA sequence as a 201 × 4 matrix where each column corresponds to a DNA letter (A, C, G or T), and for instance, a value of one in the first column corresponds to a letter A in the sequence at a given position. For one-hot encoding, colored cells indicate ones, while white cells indicate zeroes.

DeepG4 is a feedforward neural network composed of several layers illustrated in Fig 1. DNA sequence is first encoded as a one-hot encoding layer. Then, a 1-dimension convolutional layer is used with kernels to model DNA motifs. A local average pooling layer is next used. Then, the global max pooling layer extracts the highest signal from the sequence. Dropout is used for regularization. A dense layer then combines the different kernels and the activation sigmoid layer allows to compute the score between 0 and 1 of a sequence to be an active G4. The model is described in details in Subsection Results and Discussion, Deep learning approach.

Best hyperparameters including the number of kernels (900), kernel size (20 bp), kernel activation (relu), pool size (12 bp), drop-out (0%), epoch number (20), number of neurons in the dense layer (100) and the optimizer choice (rmsprop) were selected by Bayesian optimization [34]. In S1 Fig, we illustrated how changing the hyper-parameters influenced the accuracy.

### DNA motifs from DeepG4

The first layer of DeepG4 contains kernels capturing specific sequence patterns similar to DNA motifs. In order to obtain DNA motifs from the first layer (convolutional layer) of DeepG4, we proceeded as follows (see S2 Fig). For a given kernel, we computed activation values for each positive sequence. If a positive sequence contained activation values above 0 (motif hits), we extracted the sub-sequence having the maximum activation value (best motif hit sequence). The set of sub-sequences was then used to obtain a position frequency matrix (PFM) by computing the frequency of each DNA letter at each position for the kernel.

Each kernel PFM was then trimmed by removing low information content positions at each side of the PFM (threshold >0.9). PFMs whose size were lower than 5 bases after trimming were removed. PWMs were next computed from PFMs assuming background probability of 0.25 for each DNA letter as done in JASPAR.

Because many PWMs from DeepG4 were redundant, we used the motif clustering program matrix-clustering from RSAT suite (http://rsat.sb-roscoff.fr/) with parameters: median, cor = 0.6, ncor = 0.6. We used PWM cluster centers as DNA motifs for further analyses.

### DeepG4 implementation and sequence availabity

DeepG4 was implemented using Keras R library (https://keras.rstudio.com/). DeepG4 is available at https://github.com/morphos30/DeepG4. All fasta files used for training and predictions were also deposited.

### Performance analyses of DeepG4 and DeepG4*

Performance analyses of DeepG4 and DeepG4* presented in this article can be obtained using a pipeline and a docker available at https://github.com/morphos30/DeepG4ToolsComparison.

## Results and discussion

### Deep learning approach

**Fig 2:**
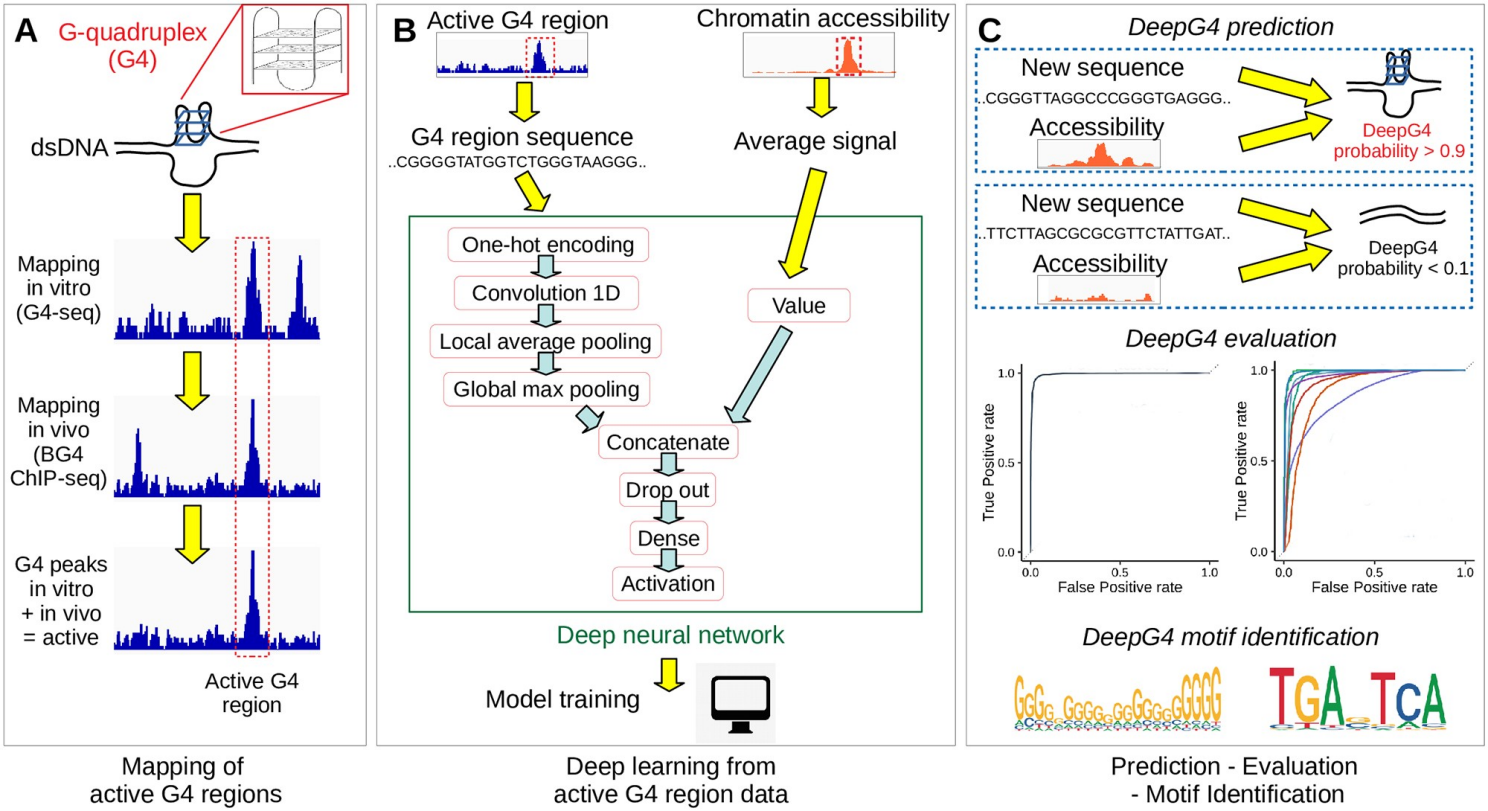
Illustration of DeepG4. A) Mapping of active G4 region sequences both in vitro and in vivo using NGS techniques. B) Deep learning model training using active G4 regions and control sequences. C) G4 activity prediction, evaluation and motif identification.

Our computational approach, called DeepG4, for predicting active G4 regions is schematically illustrated in Fig 2. In the first step (Fig 2A), we retrieved recent genome-wide mapping of in vitro G4 peak human sequences using G4-seq data [23] and of in vivo G4 peak human sequences using G4 ChIP-seq data [24]. Both methods mapped G4 regions at the resolution of few hundred base pairs, within which the exact locations of the G4s are unknown. By overlapping G4 ChIP-seq peaks with G4-seq peaks, we could identify a set of G4 peaks that were formed both in vitro and in vivo, and which we considered as “active G4 regions”. Moreover, we retrieved accessibility mapping data (DNase-seq / ATAC-seq) for the corresponding regions from the same cell line as the G4 ChIP-seq data.

In the second step (Fig 2B), we extracted the DNA sequences from active G4 regions (positive sequences). As negative sequences, we used sequences randomly drawn from the human genome with sizes, GC, and repeat contents similar to those of positive DNA sequences. For both positive and negative sequences, we computed the corresponding average chromatin accessibilities. Positive and negative sequences, together with average chromatin accessibility values, were then used to train our deep learning classifier called DeepG4. DeepG4 is a feedforward neural network composed of several layers. The DNA sequence (left input) is first encoded as a one-hot encoding layer. Then, a 1-dimension convolutional layer is used with 900 kernels (also called filters) and a kernel size of 20 bp to capture weighted DNA motifs predictive of active G4 regions. The optimal number of kernels and kernel size were determined by Bayesian optimization. A local average pooling layer with a pool size of 12 bp is next used (pool size selected by Bayesian optimization). This layer is important: it allows to aggregate kernel signals that are contiguous along the sequence, such that a G4 sequence can be modeled as multiple contiguous small motifs containing stretches of Gs. For instance, a G4 sequence can be defined by two contiguous motifs GGGNNNGGG separated by 5 bases, yielding the canonical motif GGGNNNGGGNNNNNGGGNNNGGG. Then, the global max pooling layer extracts the highest signal from the sequence for each kernel, and is concatenated with the average chromatin accessibility value (right input). Dropout is used for regularization. A dense layer then combines the different kernel signals. The activation sigmoid layer allows to compute the score between 0 and 1 of a sequence to be an active G4 region.

In the third step (Fig 2C), we used DeepG4 to predict the G4 region activity (score between 0 and 1) for a novel DNA sequence and its corresponding chromatin accessibility. We split the sequence set (set of positive and negative sequences) from HaCaT cell line (from GEO GSE76688 accession) into a training set to learn model parameters, a validation set to optimize hyper-parameters by Bayesian optimization and a testing set to assess model prediction accuracy. For this purpose, we computed the receiver operating characteristic (ROC) curve and the area under the ROC (AUROC), as well as the precision-recall (PR) curve and the area under the PR (AUPR). DeepG4 motifs are extracted from the convolutional layer.

### G4 predictions with DeepG4

**Fig 3:**
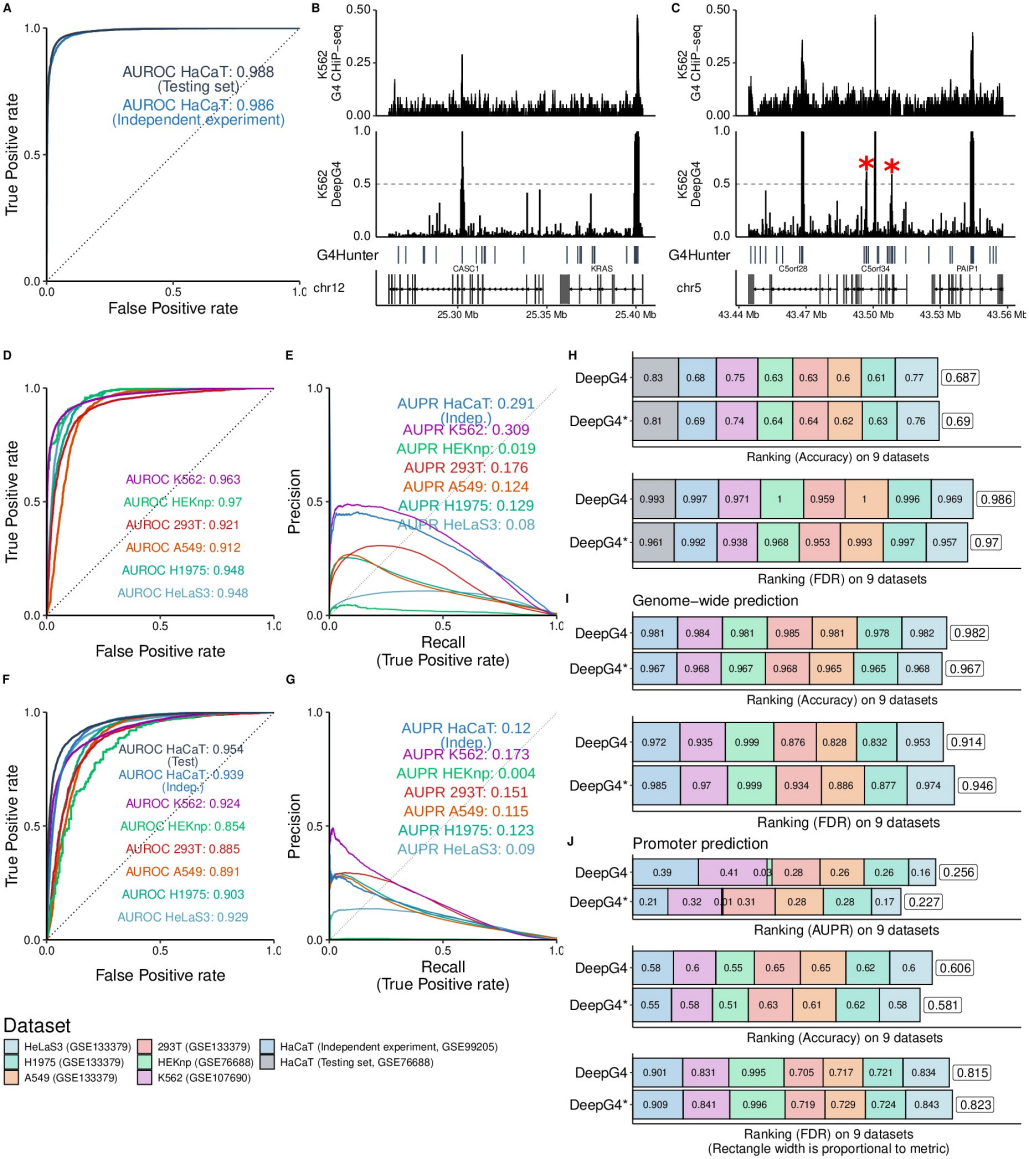
Prediction performance of DeepG4 to predict active G4 regions (regions where G4s form both in vitro and in vivo). A) Prediction performance of DeepG4. The model was trained and evaluated using HaCaT cell data. Predictions were evaluated on the testing set of sequences (same experiment as training set), but also on an independent set of sequences (from a different ChIP-seq experiment). Receiver operating characteristic (ROC) curve and area under the ROC curve (AUROC) were plotted. B) Genome browser of HaCaT-trained DeepG4 predictions and G4 ChIP-seq around KRAS gene in K562 cells. C) Genome browser of HaCaT-trained DeepG4 predictions and G4 ChIP-seq around C5orf34 gene in K562 cells. D) Prediction performance of DeepG4 trained using HaCaT data and evaluated on other cell lines. E) Genome-wide prediction performance of DeepG4 trained using HaCaT data and evaluated on other cell lines. Predictions are computed for every 200-b bins of the genome. Area Under the Precision-Recall curve is plotted (AUPR). F) Prediction performance of DeepG4* trained using HaCaT data and evaluated on other cell lines. DeepG4* is identical to DeepG4 except that chromatin accessibility is not used as input. G) Genome-wide prediction performance of DeepG4* trained using HaCaT data and evaluated on other cell lines. H) Comparison of DeepG4 and DeepG4* prediction performances, in terms of accuracy and false discovery rate (FDR) metrics. I) Comparison of DeepG4 and DeepG4* genome-wide prediction performances, in terms of accuracy and false discovery rate (FDR) metrics. J) Comparison of DeepG4 and DeepG4* promoter prediction performances, in terms of AUPR, accuracy and false discovery rate (FDR) metrics.

We then evaluated the prediction performance of DeepG4. In term of AUROC, DeepG4 obtained excellent predictions of active G4 regions from HaCaT cells on the testing set (Fig 3A; AUROC = 0.988). On an independent ChIP-seq experiment done with the same cell line (from GEO GSE99205 accession), prediction performance of DeepG4 also showed very high accuracy (AUC = 0.986; Fig 3A). We then evaluated the ability of DeepG4 trained on one cell line (HaCaT) to predict G4s in another cell line (*e.g*. K562). We first browsed the genome where G4 regions were mapped by ChIP-seq as active in K562. For instance, we looked around the oncogene KRAS known to be regulated by a G4 in its promoter (Fig 3B). ChIP-seq mapped one active G4 region in the promoter of KRAS, which was also predicted with high score by DeepG4 (score > 0.95). On the left side of KRAS, another active G4 region was mapped experimentally within CASC1 gene and was also predicted by DeepG4. On another locus, ChIP-seq mapped three main active G4 regions, located inside the genes C5orf28 (TMEM267), C5orf34 and PAIP1 (Fig 3C). These three regions were also predicted as active G4 regions with high score (score > 0.95). DeepG4 also mistakenly predicted with medium score two other regions within C5orf34 (score ≈ 0.6, red stars), which were not mapped by ChIP-seq.

Overall, DeepG4, which was trained using HaCaT cell line data, could well predict in other cell lines. For instance, the AUROC was very high for HEKnp (AUROC = 0.97; Fig 3D). For K562, HeLaS3 and H1975, AUROCs were also very good (K562: AUROC = 0.963; HeLaS3: AUROC = 0.948; H1975: AUROC = 0.948), except for 293T and A549, which presented good but slightly lower accuracy (293T: AUROC = 0.921; A459: AUROC = 0.912). We then evaluated predictions over the whole genome in an unbiased way. For this purpose, we split the genome into 200-base bins, and evaluated DeepG4 ability to discriminate between bins corresponding to active G4 regions (tens of thousands of bins) and other bins (millions of bins). Despite this highly imbalanced data, DeepG4 showed good prediction accuracy as measured by AUPR for HaCaT (AUPR = 0.291, independent experiment), K562 (AUPR = 0.309), 293T (AUPR = 0.176), A549 (AUPR = 0.124) and H1975 (AUPR = 0.129) (Fig 3E). For some cell lines, predictions were less good (HEKnp: AUPR = 0.019; HeLaS3: AUPR = 0.08).

We previously hypothesized that chromatin accessibility could help to produce cell-type specific predictions. To verify this assumption, chromatin accessibility was removed from DeepG4 model (yielding an alternative model called DeepG4*). Removing chromatin accessibility significantly lowered cell-type specific prediction accuracy. For instance, the AUROC of HaCaT (independent) was 0.939 for DeepG4* as compared to 0.986 for DeepG4, which represented an important difference (Fig 3F). We also found a large difference for HEKnp (DeepG4*, AUROC = 0.854; DeepG4, AUROC = 0.970). In terms of accuracy and false discovery rate (FDR) metrics, DeepG4* performed slightly less well than DeepG4 (Fig 3H). Regarding genome-wide predictions, removing chromatin accessibility also significantly lowered prediction performance (Fig 3G). For instance, for HaCaT (independent), we obtained an AUPR of 0.120 with DeepG4* and an AUPR of 0.291 with DeepG4. Regarding accuracy metric, DeepG4* performed less well than DeepG4, but slightly better in term of FDR (Fig 3I). We also assessed predictions on promoters to distinguish the promoters with active G4 regions from the promoters without active G4 regions. DeepG4* performed less well than DeepG4 in term of AUPR and accuracy, but slightly better in term of FDR (Fig 3J).

These results thus demonstrated the ability of DeepG4 to accurately predict cell-type specific active G4 regions from DNA sequences and chromatin accessibility. Moreover, results also revealed the importance of incorporating chromatin accessibility into DeepG4 for cell-type specific predictions.

### Identification of important motifs from DeepG4

The first layer of DeepG4 convolutional neural network encapsulated kernels that encoded DNA motifs predictive of active G4s. Hence, we extracted from the first layer the kernels and converted them to DNA motif PWMs to better understand which motifs were the best predictors of G4 activity. DeepG4 identified 900 motifs, many of them were redundant. To remove redundancy, we clustered the motifs using RSAT matrix-clustering program and kept the cluster motifs (also called root motifs in the program) for subsequent analyses. Cluster motifs could be divided into two groups: a group of de novo motifs and a group of motifs that resembled known TFBS motifs. To distinguish between these two groups, we used TomTom program (MEME suite) which mapped the cluster motifs to JASPAR database. DeepG4 motifs matching JASPAR were considered as known TFBS motifs, while motifs that did not match were classified as de novo motifs.

**Fig 4:**
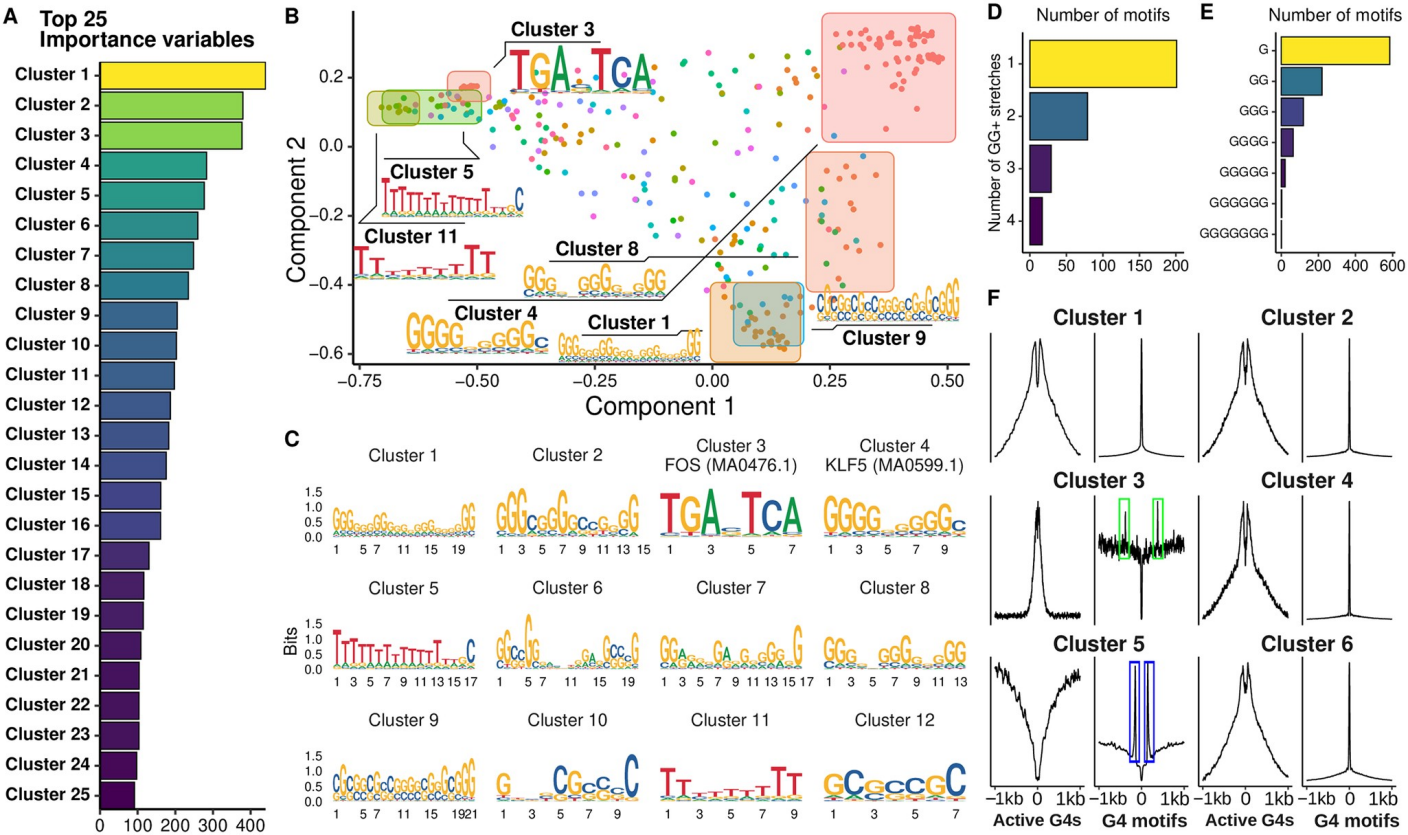
DNA motifs identified by DeepG4. A) Variable importances of DeepG4 cluster motifs, as estimated by random forests. Clustering of DeepG4 kernel motifs was done by RSAT matrix-clustering program to obtain cluster motifs. B) Multidimensional scaling (MDS) of DeepG4 motifs. As an input, matrix-clustering correlation matrix between kernel motifs was used. C) Logos of cluster motifs with highest variable importances. D) Number of kernel motifs containing one or more GG+ stretches. A GG+ stretch is defined as a stretch of 2 or more Gs in the motif consensus sequence. E) Number of kernel motifs containing G stretches depending on stretch length. F) Average profiles measuring the enrichment of cluster motifs centered around active G4 regions or canonical G4 motifs.

We first assessed the ability of DeepG4 motifs to predict active G4 regions. Hence, we computed DeepG4 cluster motif variable importances using random forests and found strong predictors (Fig 4A). In order to visualize the cluster motifs on a map, we used multi-dimensional scaling (MDS), where we also plotted the original kernel motifs used to build the cluster motifs. We found that the first MDS component reflected the guanine stretch length (higher at the right side), while the second component represented the G content (higher at the bottom) (Fig 4B).

Many strong predictors were de novo motifs which ressembled the G4 canonical motif or parts of the canonical motif. For instance, cluster 1 comprised 4 stretches of GG+, thus almost forming a canonical G4 motif (Fig 4C). Cluster 2 comprised three stretches of GG+, could thus be considered as three quarters of a canonical G4 motif. We then counted GG+ stretches (stretches of 2 or more guanines) from the kernel motifs and found that many kernel motifs contained more than one GG+ stretch (Fig 4D). Moreover, the guanine stretches were of varying lengths, ranging from one G up to 5 Gs (Fig 4E). Among the best predictors, we also found several motifs corresponding to known TFBS motifs (Fig 4C). For instance, the third best predictor, cluster 3, almost perfectly matched FOS motif MA0476.1 (q-value = 2 × 10^−10^). Other strong predictors, such as cluster 4, matched KLF5 motif MA0599.1 (q-value = 0.09). It was very interesting to observe that such motif corresponding to one half of a canonical G4 motif also matched a known TFBS motif, which supported the complex interplay between G4s and TFBS protein binding [35].

We then assessed the enrichment of DeepG4 cluster motifs around active G4 regions and around canonical G4 motifs (Fig 4F). Motifs ressembling G4 canonical motif or parts of it, such as clusters 1 and 2, were enriched at both active G4 regions and canonical G4 motifs, thus representing actual G4 structures. But other motifs that were very different from the G4 canonical motif, such as cluster 3, were strongly enriched at active G4 regions, but depleted at the exact location of canonical G4 motifs. Interestingly, cluster 3 was enriched close to the canonical G4 motifs (around 300 bp, framed in green), suggesting that cluster 3 (FOS motif MA0476.1) did not participate directly to the G4 structure, but could act in the vicinity to support G4 activity. Conversely, we also found a motif composed mainly of Ts (poly(T) tract), the cluster 5 motif, which was depleted in active G4 regions, but which was at the same time enriched in the vicinity of canonical G4 motifs (framed in blue). This suggests that such poly(T) motif could inhibit the activity of G4 motifs by acting in the vicinity.

These observations revealed the important role of TFBS motifs that could act directly in G4 activity as part of G4 structure, as previously shown for SP1 in vitro [36], or could participate indirectly to support or inhibit G4 activity in the vicinity of G4s such as FOS motif (AP-1 complex).

### Genome-wide predictions in tissues and cancers

Using DeepG4, we could map active G4 regions genome-wide in many different tissues and cancers for which no G4 ChIP-seq experiments were available, but for which we could find publicly available chromatin accessibility data (ATAC-seq or DNase-seq). Hence, we made the mapping available on the DeepG4 Github repository as a resource for the G4 community.

**Fig 5:**
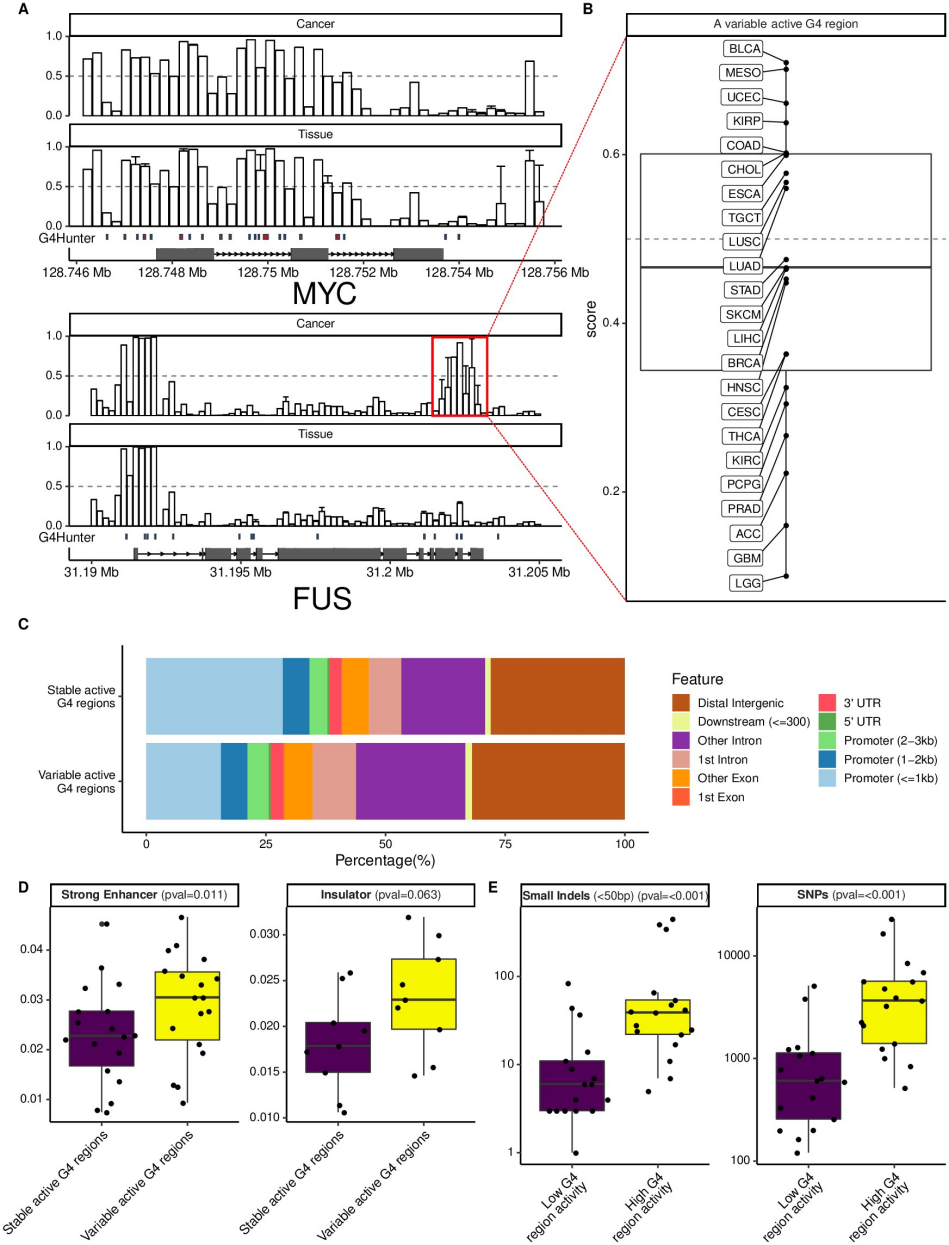
Genome-wide prediction of active G4 regions in tissues and cancers. A) Genome browser of DeepG4 predictions at MYC and FUS genes in tissues and cancers. B) Relationship between DeepG4 predicted G4 activity and the amount of mutations, depending on the mutation class. Cancer cohort abbreviations (*e.g*. MESO) are detailed in S1 Table. C) Annotations of predicted stable and variable active G4 regions. D) Mutation rates in BRCA breast cancer depending on predicted G4 region activity.

We first browsed the genome at known oncogenes and looked at predicted active G4 regions (Fig 5A). In MYC, we predicted many active G4 regions in the promoter but also in the exons and introns. Predicted G4 activity was rather stable and did not vary across the tissues and cancers. In another gene, FUS, we found that the promoter contained an active G4 region that was very stable across tissues and cancer (left side), but we also could identify another G4 region toward the transcription end site (TES, right side) that was not predicted to be active in tissues, but predicted to be active in some cancers (framed in red), in particular in MESO (Mesothelioma), UCEC (Uterine Corpus Endometrial Carcinoma) and BLCA (Bladder Cancer), and inactive in some other cancers including GBM (Brain Cancer) and LGG (Brain Lower Grade Glioma) (Fig 5B). Thus, DeepG4 could identify regions of variable G4 activity. Overall, only a minority of predicted G4 regions varied across the tissues and cancers (around 10%). When we annotated these regions and compared with stable G4 regions, we observed that 29% of stable G4 regions located within promoters, whereas only 16% of variable G4 regions colocalized with promoters (Fig 5C). Instead, we found variable G4 regions in intronic and intergenic regions. We further explored the role of variable G4 regions by using annotations from ENCODE in multiple cell lines from ChromHMM tool [33]. We found that variable G4 regions were enriched at strong enhancers as compared to stable G4 regions (*p* = 0.011, Fig 5D), and we also found a near-significant enrichment at insulator regions (*p* = 0.063, Fig 5D) in agreement with previous studies showing enrichment near CTCF at 3D domain (topologically associating domain, TAD) borders [37].

Since G4s are known mutagenic regions when unresolved, we then looked at the link between G4 activity and mutation rates in BRCA breast cancer (Fig 5E). We found a strong positive link between high G4 activity and SNP and small indel mutation rates, meaning that when G4s were formed in vivo they had a higher chance of yielding mutations and therefore this suggests that the chromatin landscape could greatly influence G4 impact on genome instability at a local scale.

## Conclusion

In this article, we propose a novel deep learning method, named DeepG4, to predict active G4 regions from DNA sequence and chromatin accessibility. The proposed method is designed to predict active G4 regions *i.e*. regions that are detected both in vitro and in vivo, unlike previous algorithms that were developed to predict G4s forming in vitro (naked DNA). For this purpose, our method exploits the genomic context of G4s, which comprises the G4(s) as well as other motifs in the vicinity that may play a role in G4 activity (*i.e*. transcription factor motifs). Moreover, adding chromatin accessibility into the model allows to predict active G4 regions depending on the cell type. Our novel method which maps active G4 regions in a cell-type specific manner at 201-bp resolution is complementary to existing algorithms based on regular expression (*e.g*. quadparser) and scores (*e.g*. G4Hunter), which map the exact location of potential G4 forming sequences and propensities. Moreover, DeepG4 provides a useful tool for mapping active G4 regions for cell lines, tissues and cancers for which no experimental data are available to date. Therefore, DeepG4 comprehensive predictions in tissues and cancers will represent a useful resource for the G4 community.

DeepG4 uncovered numerous specific DNA motifs predictive of active G4s. Many motifs resembled the canonical G4 motif (*G*_3+_ *N*_1−7_ *G*_3+_ *N*_1−7_ *G*_3+_ *N*_1−7_ *G*_3+_) or even parts of it. Most notably, many motifs corresponded to half or 3/4 of the canonical motif. The combination of these G4 parts, which is captured by DeepG4 as a deep neural network, brings flexibility in G4 modeling. Strikingly, some motifs completely or partly matched known TFBS motifs including KLF5 motif MA0599.1 and FOS (AP-1) motif MA0476.1, suggesting that they could contribute directly to G4 structures themselves or participate indirectly in G4 activity in the vicinity through the binding of transcription factors. In line with this result, it was previously found that G4s are enriched in the vicinity of the architectural protein CTCF at 3D domain (topologically associating domain, TAD) borders [37]. Moreover, it has been shown that SP1 binds to G4s with a comparable affinity as its canonical motif [36], and that G4s are TF hubs [35]. It was also surprising to find a poly(T) motif (cluster 5 motif) depleted in active G4 regions but enriched in the vicinity of canonical G4 motifs, suggesting that such motif could inhibit the activity of canonical G4 motifs in its vicinity.

In addition, we used DeepG4 to predict active G4 regions genome-wide in many tissues and cancers, thereby providing a resource for the chromatin and G4 community. Interestingly, we identified two types of active G4 regions, those stable across tissues and cancers, and those less frequent that are variable. We found that variable active G4 regions are located within intronic and intergenic regions, and could act as enhancers and insulators, unlike stable G4 regions that are more enriched in promoters.

There are several limitations of the proposed approach. First, one limit of DeepG4 (as well as the other existing machine/deep learning methods) is that it requires a region of several hundred bases, thereby restricting the resolution of G4 mapping. Once an active G4 region is mapped, methods such as G4Hunter or pqsfinder have to be used to identify the exact position of the G4(s) within the region. Our model could be improved by adding novel neural layers in order to find as well the exact location of potential G4 sequences. Second, DeepG4 does not process the DNA sequence in a strand-specific manner, thus a given motif could be redundantly encoded in both strands within the convolutional layer. However, post-processing of DeepG4 motifs using methods such as matrix-clustering alleviates such problem by mapping complementary motifs (same motifs on different strands) to each other to merge them into cluster motifs. Third, the prediction performance of DeepG4 strongly depends on existing datasets that are limited, potentially inaccurate and biased, especially regarding in vivo mapping. Once more techniques for in vivo G4 mapping will be developped, DeepG4 will need to be retrained in order to improve prediction accuracy. Moreover, since DeepG4 was trained based on human data, predictions on non-mammalian genomes are expected to be less accurate. Fourth, DeepG4 is limited to predict active G4s but a similar approach could be used to predict any active non-B DNA structure using permanganate/S1 nuclease footprinting data [38].

## Supporting information

S1 Fig Prediction accuracy estimated from the validation set depending on hyper-parameters, as found from Bayesian optimization. For each hyper-parameter, the optimum is marked as a red triangle.
(TIF)

S2 Fig Extraction and processing of DNA motifs from DeepG4 convolutional layer. (TIF)

S1 Table Cancer cohort abbreviations from ICGC project. (TIF)

## References

[1] Watson JD, Crick FH. A structure for deoxyribose nucleic acid. Nature. 1953;171:737–738. doi: 10.1038/171737a0 13054692

[2] Sen D, Gilbert W. Formation of parallel four-stranded complexes by guanine-rich motifs in DNA and its implications for meiosis. Nature. 1988;334(6180):364–366. doi: 10.1038/334364a0 3393228

[3] Chen Y, Yang D. Sequence, stability, and structure of G-quadruplexes and their interactions with drugs. Current Protocols in Nucleic Acid Chemistry. 2012;50(1):17.5.1–17.5.17. 10.1002/0471142700.nc1705s50 PMC3463244 22956454

[4] Bhattacharyya D, Mirihana Arachchilage G, Basu S. Metal cations in G-quadruplex folding and stability. Frontiers in Chemistry. 2016;4:38. 27668212 10.3389/fchem.2016.00038 PMC5016522

[5] Spiegel J, Adhikari S, Balasubramanian S. The structure and function of DNA G-quadruplexes. Trends in Chemistry. 2019;. 10.1016/j.trechm.2019.07.002 PMC7472594 32923997

[6] Fay MM, Lyons SM, Ivanov P. RNA G-quadruplexes in biology: Principles and molecular mechanisms. Journal of Molecular Biology. 2017;429(14):2127–2147. doi: 10.1016/j.jmb.2017.05.017 28554731 PMC5603239

[7] Varshney D, Spiegel J, Zyner K, Tannahill D, Balasubramanian S. The regulation and functions of DNA and RNA G-quadruplexes. Nature Reviews Molecular Cell Biology. 2020;21(8):459–474. doi: 10.1038/s41580-020-0236-x 32313204 PMC7115845

[8] Sfeir A. Telomeres at a glance. Journal of Cell Science. 2012;125(18):4173–4178. doi: 10.1242/jcs.106831 23135002 PMC6518153

[9] Wang Q, Liu Jq, Chen Z, Zheng Kw, Chen Cy, Hao Yh, et al. G-quadruplex formation at the 3’ end of telomere DNA inhibits its extension by telomerase, polymerase and unwinding by helicase. Nucleic Acids Research. 2011;39(14):6229–6237. doi: 10.1093/nar/gkr164 21441540 PMC3152327

[10] Bryan TM. G-quadruplexes at telomeres: Friend or foe? Molecules. 2020;25(16). doi: 10.3390/molecules25163686 32823549 PMC7464828

[11] Brooks TA, Hurley LH. Targeting MYC expression through G-quadruplexes. Genes & Cancer. 2010;1(6):641–649. doi: 10.1177/1947601910377493 21113409 PMC2992328

[12] Marnef A, Cohen S, Legube G. Transcription-coupled DNA double-strand break repair: Active genes need special care. Journal of Molecular Biology. 2017;429(9):1277–1288. doi: 10.1016/j.jmb.2017.03.024 28363678

[13] Cimino-Reale G, Zaffaroni N, Folini M. Emerging role of G-quadruplex DNA as target in anticancer therapy. Current Pharmaceutical Design. 2016;22(44):6612–6624. 27587203 10.2174/1381612822666160831101031

[14] Asamitsu S, Takeuchi M, Ikenoshita S, Imai Y, Kashiwagi H, Shioda N. Perspectives for applying G-quadruplex structures in neurobiology and neuropharmacology. International Journal of Molecular Sciences. 2019;20(12). doi: 10.3390/ijms20122884 31200506 PMC6627371

[15] Hänsel-Hertsch R, Simeone A, Shea A, Hui WWI, Zyner KG, Marsico G, et al. Landscape of G-quadruplex DNA structural regions in breast cancer. Nature Genetics. 2020;52(9):878–883. doi: 10.1038/s41588-020-0672-8 32747825

[16] International Human Genome Sequencing Consortium. Initial sequencing and analysis of the human genome. Nature. 2001;409(6822):860–921. doi: 10.1038/35057062 11237011

[17] Puig Lombardi E, Londono-Vallejo A. A guide to computational methods for G-quadruplex prediction. Nucleic Acids Research. 2019;48(1):1–15. 10.1093/nar/gkz1097 PMC6943126 31754698

[18] Miskiewicz J, Sarzynska J, Szachniuk M. How bioinformatics resources work with G4 RNAs. Briefings in Bioinformatics. 2020;. 10.1093/bib/bbaa201 PMC8138894 32898859

[19] Huppert JL, Balasubramanian S. Prevalence of quadruplexes in the human genome. Nucleic Acids Research. 2005;33(9):2908–2916. doi: 10.1093/nar/gki609 15914667 PMC1140081

[20] Huppert JL, Balasubramanian S. G-quadruplexes in promoters throughout the human genome. Nucleic Acids Research. 2006;35(2):406–413. 17169996 10.1093/nar/gkl1057 PMC1802602

[21] Bedrat A, Lacroix L, Mergny JL. Re-evaluation of G-quadruplex propensity with G4Hunter. Nucleic Acids Research. 2016;44(4):1746–1759. doi: 10.1093/nar/gkw006 26792894 PMC4770238

[22] Hon J, Martinek T, Zendulka J, Lexa M. pqsfinder: an exhaustive and imperfection-tolerant search tool for potential quadruplex-forming sequences in R. Bioinformatics. 2017;33(21):3373–3379. doi: 10.1093/bioinformatics/btx413 29077807

[23] Chambers VS, Marsico G, Boutell JM, Di Antonio M, Smith GP, Balasubramanian S. High-throughput sequencing of DNA G-quadruplex structures in the human genome. Nature Biotechnology. 2015;33(8):877–881. doi: 10.1038/nbt.3295 26192317

[24] Hänsel-Hertsch R, Beraldi D, Lensing SV, Marsico G, Zyner K, Parry A, et al. G-quadruplex structures mark human regulatory chromatin. Nature Genetics. 2016;48(10):1267–1272. doi: 10.1038/ng.3662 27618450

[25] Sahakyan AB, Chambers VS, Marsico G, Santner T, Di Antonio M, Balasubramanian S. Machine learning model for sequence-driven DNA G-quadruplex formation. Scientific Reports. 2017;7(1):14535. doi: 10.1038/s41598-017-14017-4 29109402 PMC5673958

[26] Klimentova E, Polacek J, Simecek P, Alexiou P. PENGUINN: Precise exploration of nuclear G-quadruplexes using interpretable neural networks. bioRxiv. 2020;. doi: 10.3389/fgene.2020.568546 33193663 PMC7653191

[27] Barshai M, Orenstein Y. Predicting G-quadruplexes from DNA sequences using multi-kernel convolutional neural networks. In: Proceedings of the 10th ACM International Conference on Bioinformatics, Computational Biology and Health Informatics. BCB’19. New York, NY, USA: Association for Computing Machinery; 2019. p. 357–365. Available from: 10.1145/3307339.3342133.

[28] Hänsel-Hertsch R, Spiegel J, Marsico G, Tannahill D, Balasubramanian S. Genome-wide mapping of endogenous G-quadruplex DNA structures by chromatin immunoprecipitation and high-throughput sequencing. Nature Protocols. 2018;13(3):551–564. doi: 10.1038/nprot.2017.150 29470465

[29] Mao SQ, Ghanbarian AT, Spiegel J, Martínez Cuesta S, Beraldi D, Di Antonio M, et al. DNA G-quadruplex structures mold the DNA methylome. Nature Structural & Molecular Biology. 2018;25(10):951–957. doi: 10.1038/s41594-018-0131-8 30275516 PMC6173298

[30] Zheng Kw, Zhang Jy, He Yd, Gong Jy, Wen Cj, Chen Jn, et al. Detection of genomic G-quadruplexes in living cells using a small artificial protein. Nucleic Acids Research. 2020;48(20):11706–11720. doi: 10.1093/nar/gkaa841 33045726 PMC7672459

[31] The ENCODE Consortium. An integrated encyclopedia of DNA elements in the human genome. Nature. 2012;489(7414):57–74. doi: 10.1038/nature11247 22955616 PMC3439153

[32] Zhang J, Bajari R, Andric D, Gerthoffert F, Lepsa A, Nahal-Bose H, et al. The International Cancer Genome Consortium Data Portal. Nature Biotechnology. 2019;37(4):367–369. doi: 10.1038/s41587-019-0055-9 30877282

[33] Ernst J, Kellis M. ChromHMM: automating chromatin-state discovery and characterization. Nature Methods. 2012;9(3):215–216. doi: 10.1038/nmeth.1906 22373907 PMC3577932

[34] Snoek J, Larochelle H, Adams RP. Practical Bayesian optimization of machine learning algorithms. In: Proceedings of the 25th International Conference on Neural Information Processing Systems—Volume 2. NIPS’12. Red Hook, NY, USA: Curran Associates Inc.; 2012. p. 2951–2959.

[35] Spiegel J, Cuesta SM, Adhikari S, Hänsel-Hertsch R, Tannahill D, Balasubramanian S. G-quadruplexes are transcription factor binding hubs in human chromatin. Genome Biology. 2021;22(1):117. doi: 10.1186/s13059-021-02324-z 33892767 PMC8063395

[36] Raiber EA, Kranaster R, Lam E, Nikan M, Balasubramanian S. A non-canonical DNA structure is a binding motif for the transcription factor SP1 in vitro. Nucleic Acids Research. 2011;40(4):1499–1508. 22021377 10.1093/nar/gkr882 PMC3287196

[37] Hou Y, Li F, Zhang R, Li S, Liu H, Qin ZS, et al. Integrative characterization of G-Quadruplexes in the three-dimensional chromatin structure. Epigenetics. 2019;14(9):894–911. doi: 10.1080/15592294.2019.1621140 31177910 PMC6691997

[38] Kouzine F, Wojtowicz D, Baranello L, Yamane A, Nelson S, Resch W, et al. Permanganate/S1 nuclease footprinting reveals non-B DNA structures with regulatory potential across a mammalian genome. Cell Systems. 2017;4(3):344–356.e7. doi: 10.1016/j.cels.2017.01.013 28237796 PMC7432990

