# Supplementary figures and images for: DeepG4: A deep learning approach to predict cell-type specific active G-quadruplex regions
Source: PLoS Comput Biol. 2021 Aug 12;17(8):e1009308. doi: 10.1371/journal.pcbi.1009308 (PMC8384162; doi:10.1371/journal.pcbi.1009308)

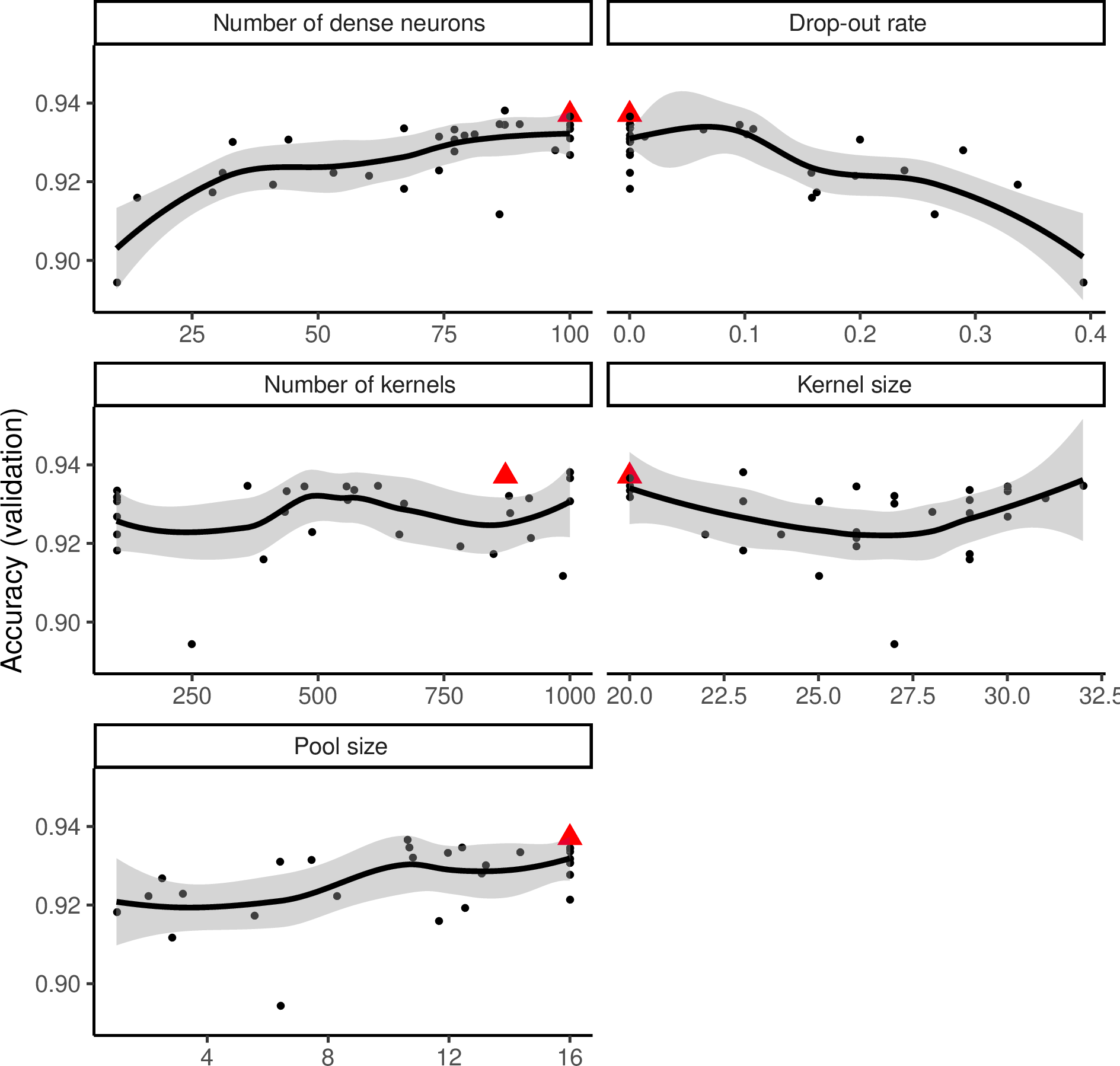

Supplement: S1 Fig — For each hyper-parameter, the optimum is marked as a red triangle. (TIF) [file pcbi.1009308.s001.tif]

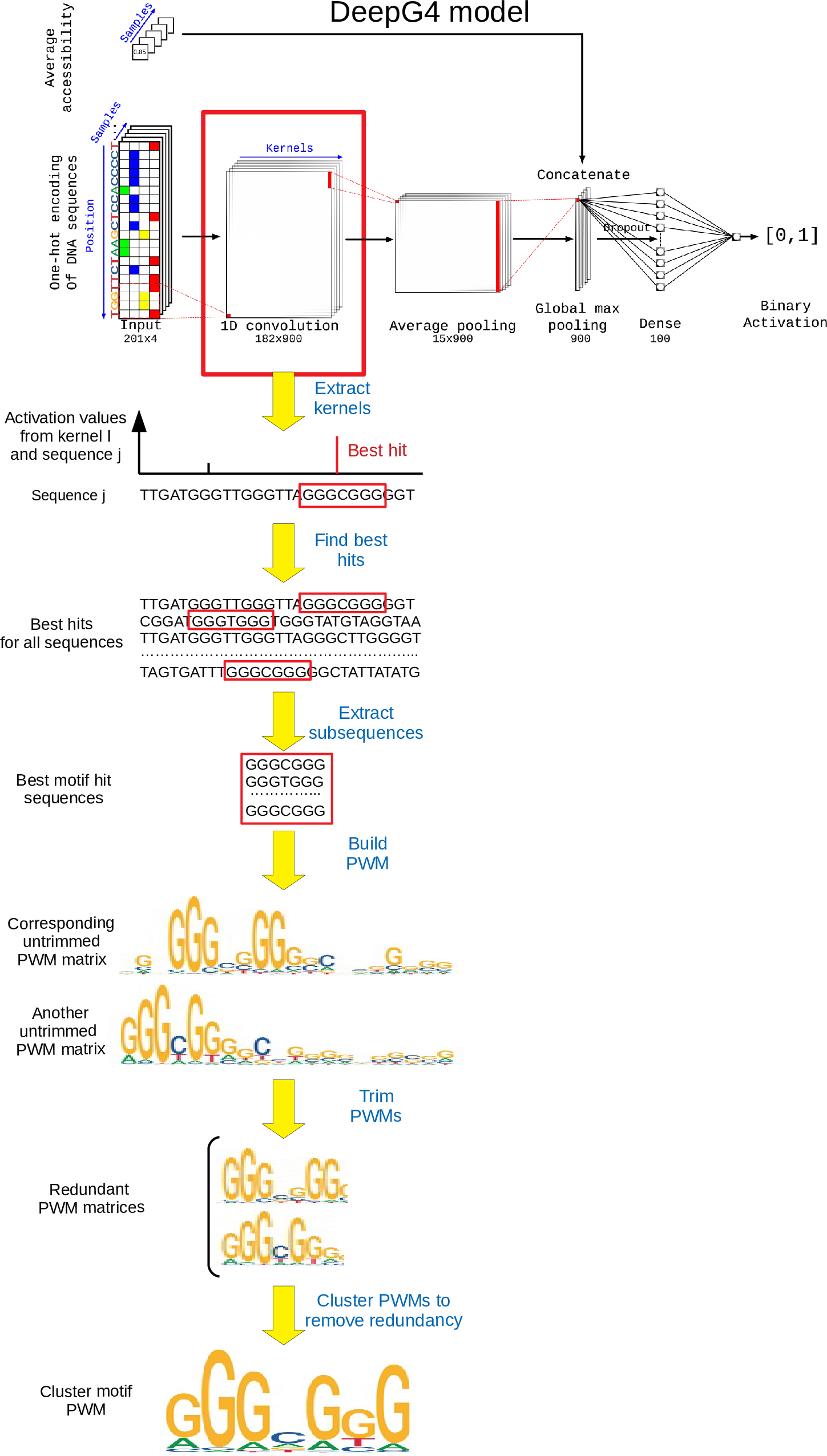

Supplement: S2 Fig — (TIF) [file pcbi.1009308.s002.tif]

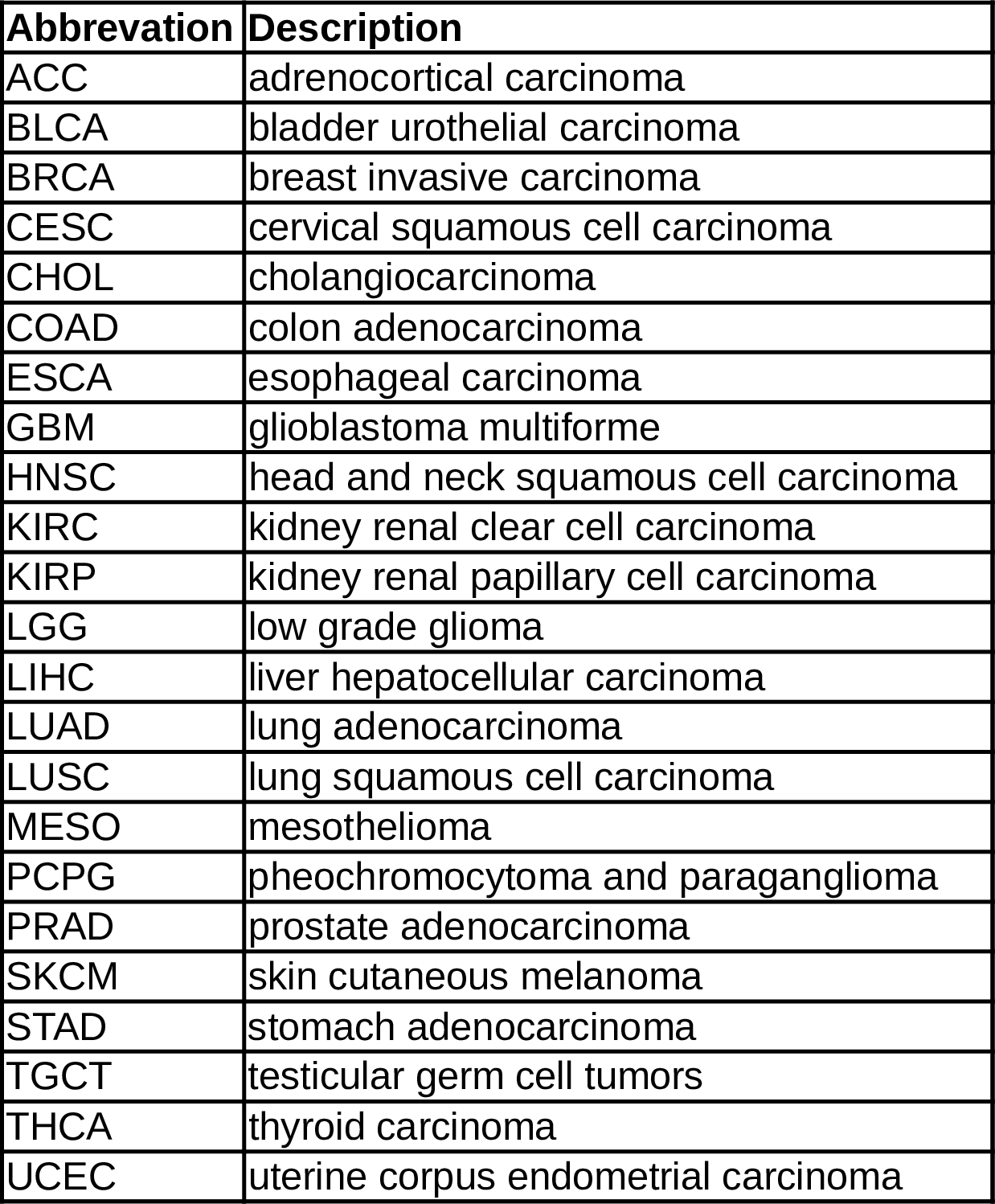

Supplement: S1 Table — (TIF) [file pcbi.1009308.s003.tif]
